# All-trans retinoic acid regulates the expression of the extracellular matrix protein fibulin-1 in the guinea pig sclera and human scleral fibroblasts

**Published:** 2010-04-15

**Authors:** Chuanxu Li, Sally A. McFadden, Ian Morgan, Dongmei Cui, Jianmin Hu, Wenjuan Wan, Junwen Zeng

**Affiliations:** 1 State Key Laboratory of Ophthalmology, Zhongshan Ophthalmic Center, Sun Yat-sen University, Guangzhou, China; 2 Vision Sciences Laboratory, School of Psychology, Faculty of Science and IT, The University of Newcastle, Australia; 3 Visual Sciences Group, Research School of Biology, Australian National University, Canberra, Australia

## Abstract

**Purpose:**

Fibulin-1 (*FBLN1*) mRNA is expressed in human sclera and is an important adhesion modulatory protein that can affect cell–matrix interactions and tissue remodeling. Scleral remodeling is influenced by all-trans retinoic acid (RA). Our purpose was to confirm the presence of fibulin-1 protein in guinea pig sclera and investigate the effect of RA on the expression of fibulin-1 in guinea pig sclera in vivo and in cultured human scleral fibroblasts (HSFs).

**Methods:**

Confocal fluorescence microscopy was used to study fibulin-1 and aggrecan expression and localization in sclera from control guinea pigs and in animals given RA by daily gavage from 4 to 8 days of age. The effects of RA (from 10^−9^ to 10^−5^ M) on fibulin-1 expression in HSFs were observed by immunohistochemistry and assayed by real-time PCR and western blot analysis.

**Results:**

Fibulin-1 protein expression was detected by confocal fluorescence microscopy in guinea pig sclera and in cultured HSFs. Upregulation of fibulin-1 in scleral tissue was observed after feeding with RA. In vitro, the level of *Fbln1* mRNA was increased after treatment of HSFs with RA (at concentrations of 10^−8^ to 10^−6^ M; p<0.001), with a maximum effect at 10^−7^ M. Fibulin-1 protein levels were significantly increased after treatment of HSFs with 10^−7^ M of RA for 24 or 48 h (p<0.05).

**Conclusions:**

Fibulin-1 protein was expressed in guinea pig sclera and cultured HSFs. Expression was regulated by RA, a molecule known to be involved in the regulation of eye growth. Further studies on the role of fibulin-1 in the regulation of eye growth, including during the development of myopia, are therefore warranted.

## Introduction

Fibulin-1 is a member of the seven-member fibulin family and is an important extracellular matrix (ECM) scaffolding protein [1]. It forms intermolecular bridges that stabilize the organization of the supramolecular ECM structure,and also plays an essential role in tissue remodeling, affecting cell adhesion, migration, proliferation, and differentiation [2]. It is required for the morphogenesis of neural crest-derived structures, such as sclera [3].

Aggrecan is a proteoglycan present in human sclera [4]. The modulation of aggrecan expression may play a key functional role in scleral remodeling [5]. Fibulin-1 is a ligand for aggrecan [6] and could therefore also be an important factor in scleral remodeling. Recent work using a human sclera cDNA library has shown that fibulin-1 (*FBLN1*) was expressed in the sclera [7]. However, the expression, location, and role of the fibulin-1 protein in sclera have not been reported.

All-trans retinoic acid (RA) is a potent regulator of cell growth and differentiation in various types of cells and can regulate ECM metabolism in many systems [8]. It is an important molecular signal in the control of eye size, perhaps acting as a signal for the direction of ocular growth [9,10]. Although the mechanism by which RA regulates ocular size is not clear, it may affect scleral ECM during scleral remodeling [10]. RA can regulate aggrecan expression and skeletal growth and articular cartilage remodeling in skeletal systems [11]. We sought here to determine whether RA affects the expression of aggrecan and fibulin-1 in guinea pig sclera. It has been shown that six RA receptor subtypes are present in cultured human scleral fibroblasts (HSFs) [12], suggesting that RA may interact with these cells. However, whether RA treatment affects the expression of fibulin-1 in HSFs has not been studied.

The purpose of this study was first to examine the localization of fibulin-1 protein in guinea pig sclera and the presence of fibulin-1 protein in cultured HSFs. Second, we examined whether the expression of fibulin-1 in guinea pig sclera (in vivo) was regulated by RA given by daily gavage [13] and in cultured HSFs treated with RA (in vitro).

## Methods

### Guinea pig tissue and housing

Pigmented guinea pigs were obtained from the the University of Newcastle, Australia. They were reared in litters with their mother until 9 days of age when scleral tissue was extracted. During rearing, animals were housed in plastic boxes (65×45×30 cm) lined with a bed of paper pellets and with stainless steel wire lids. Lighting was on a 12 h:12 h light–dark cycle and was provided by overhead incandescent lamps evenly diffused through a perspex barrier located 15 cm above the cages. The average light intensity in the cage was 300 lux. To treat the sclera with RA exogenously, during rearing, young guinea pigs were fed 0.5 ml of RA (24 mg/kg, n=8 eyes) or carrier alone (peanut oil [PO] n=4 eyes) from 4 to 8 days of age every day, 1 h after the beginning of the light cycle. Half of each litter was given either RA or PO to ensure a matched-pair design. Feeding was by gavage while the guinea pigs were lightly anesthetized with isoflurane (1.5% in O_2_). At 8 days of age, 24 h after the day 7 gavage and before the day 8 gavage, eyes were cyclopleged with 1% cyclopentolate. One hour later, refractive error was measured using streak retinoscopy followed by measurement of axial ocular dimensions in animals using A-scan ultrasonography. These measures were the same as previously described [9] and were used to confirm that the RA treatment had affected ocular elongation First, the animals were lightly anesthetized with 1.5% isoflurane in oxygen. Then, the anesthetized animals were restrained to allow alignment of the transducer probe to the center of the pupil along the optic axis. Approximately 10 measures were taken from each eye sequentially, with the probe realigned several times. On day 9, animals were euthanized by intraperitoneal overdose of barbiturate, eyes were removed on ice, and tissue extracted for immunohistochemistry. All procedures were in compliance with the New South Wales Animal Research Act and approved by the Animal Ethics Committee of the University of Newcastle, Newcastle, Australia.

### Immunohistochemistry on guinea pig sclera

To observe the expression and localization of fibulin-1 in scleral tissue and find the relationship between fibulin-1 and aggrecan, immunohistochemical staining was observed in scleras from animals fed PO or RA. On day 9 (24 h after the last gavage), eyes were removed and eye cups (without the vitreous) were fixed for 30 min at 20 °C in 4% paraformaldehyde in 0.1 M phosphate buffer, pH 7.4. Fixed samples were washed three times in PBS (0.05 M phosphate buffer, 195 mM NaCl, and 3 mM NaN_3_, pH 7.4), cryoprotected in PBS plus 30% sucrose, soaked in embedding medium (optimal cutting temperature compound) for 10 min, and freeze-mounted onto sectioning blocks. Vertical sections (10–12 μm thick) were cut from the posterior pole of the eye and thaw mounted onto gelatin-coated glass slides. Sections from RA- and PO-treated eyes were placed together in pairs on each slide to ensure equal exposure to reagents. Sections were air dried, ringed with rubber cement to form a well for antibody solutions, and stored at −20 °C until use.

### Human scleral fibroblast isolation and identification

This part of the study was approved by the Ethics Committee of Sun Yat-sen University, Guangzhou, China, and complied with the tenets of the Declaration of Helsinki for Research Involving Human Tissue. Normal human eyes (n=8) from donors ranging from 10 to 20 years of age were obtained from the Eye Bank of Zhongshan Ophthalmic Center (Sun Yat-sen University) and were used to make cultures of HSFs. Eyes were washed immediately in Hank's balanced salt solution (HBSS; Gibco, Grand Island, NY) with penicillin (200 μg/ml penicillin–streptomycin) (Invitrogen, Carlsbad, CA) and gentamicin sulfate (400 μg/ml; Invitrogen). The retinas and choroids were removed from the sclera. The posterior sclera was trimmed into 1×1-mm^2^ pieces, placed in DMEM/F12 (Gibco) with 1× antibiotic/antimycotic (penicillin–streptomycin; Invitrogen), and 10% fetal bovine serum (Gibco), and then incubated at 37 °C in a humidified incubator containing 5% CO_2_. The growth medium was changed every 3 days. When a heavy primary monolayer was achieved, cells were trypsinized for 2 min at room temperature in 0.25% trypsin/ethylene diamine tetraacetic acid (EDTA) solution in PBS (Gibco). Cells were subcultured at a split ratio of 1:3 in a 25-mm^2^ plastic bottle. The third passage of fibroblasts was used for this experiment. The fibroblasts were grown on coverslips in six-well plates (Corning Ltd., Tokyo, Japan) to 70%–80% confluence. The cells were washed with PBS three times, fixed with acetone for 15 min, air dried, and kept frozen at −20 °C until use. The purity of fibroblast cultures was confirmed by staining for vimentin and stain resistance for cytokeratin, desmin, and S-100, using the indirect immunofluorescence procedure, as previously described [14].

### All-trans retinoic acid (RA) treatment of cell cultures

RA was dissolved in dimethylsulfoxide (DMSO) to a concentration of 10^−2^ M, and this solution was diluted into DMEM/F12 to different concentrations and stored at 4 °C for immediate use or frozen in aliquots at −20 °C. Medium containing 0.1% DMSO, which is the highest final concentration used in these experiments, served as the control.

The numbers of cells in the cultures incubated with RA were estimated using the cell counting Kit-8 (Dojin Laboratories, Kumamoto, Japan) containing 2-(2-methoxy-4-nitrophenyl) −3-(4-nitrophenyl)-5-(2,4-disulfophenyl)-2H-tetrazolium (WST-8) and 1-methoxyphenazine methosulfate (1-methoxy-PMS). Briefly, HSFs were seeded into 96-well plates coated with rat tail tendon type I collagen (Sigma, St. Louis, MO) at an initial density of 10^5^ cells/well and pre-incubated with DMEM/F12 without fetal bovine serum (FBS) for 24 h. Various concentrations of RA (from 10^−9^ M to 10^−4^ M) or control medium were added. The cultures were incubated for a further 24 or 48 h. The solution of WST-8 and 1-methoxy-PMS was then added to each well, and the plates were incubated for 1 h, after which the absorbance at 450 nm was measured to determine the amount of formazan dye generated by dehydrogenases in cells, which is directly proportional to the number of living cells.

To extract total RNA or total protein for real-time PCR and western blot analysis, HSFs were plated in standard plates coated with rat tail tendon type I collagen with DMEM/F12 in 10% FBS at the same cell density for 24 h. The medium was changed to DMEM/F12 without FBS for another 24 h. Finally, these cells were grown in medium containing RA (from 10^−9^ M to 10^−5^M) or in medium containing 0.1% DMSO as an untreated control. After 24 h of culture, total RNA was extracted. In a second experiment, cells were grown in control medium or in medium with RA at concentrations of either 10^−7^ M or 10^−6^ M. Total RNA was extracted after 12 h, 24 h, or 48 h, and total protein was extracted after 24 h or 48 h. Quantitative real-time PCR analysis and western blot analysis were undertaken on extracted total RNA and homogenized total protein, respectively. After 48 h of culture, the morphology of fibroblasts was also observed with light microscopy in control medium and in medium with 10^−7^ M RA.

### Indirect immunofluorescence

Fibulin-1 was visualized using standard immunohistochemistry in guinea pig sclera from animals fed RA or PO, and in both control and RA-treated HSFs. Briefly, fixed sections or cells were washed three times with PBS, covered with 10% normal donkey serum diluted in PBS, and incubated for 20 min at 37 °C. The slides were incubated at 4 °C overnight with primary antibodies (anti-fibulin-1 diluted to 1:50 in PBS, anti-aggrecan diluted to 1:50 in PBS; Santa Cruz Biotechnology, Santa Cruz, CA). Cells and sections were incubated in PBS without primary antibodies as a negative control. The antibody-treated and negative control sample slides were washed with PBS and exposed to DyLight 488-conjugated anti-rabbit immunoglobulin G (IgG) antibodies or Dylight 594-conjugated anti-goat IgG (Jackson ImmunoResearch Laboratories Inc., West Grove, PA) at 1:1,000 in PBS at 37 °C for 60 min. The slides were washed in PBS three times, and cell nuclei were stained with Hoechst 33342 or propidium iodide (Sigma). Immunofluorescence images were taken using a confocal microscope (LSM 510 META; Carl Zeiss, Jena, Germany).

### Quantitative real-time PCR analysis

Real-time PCR analysis was used to show the expression of fibulin-1 at the transcription level. Total RNA was extracted from cultured HSFs using Trizol Reagent (Invitrogen). Complemenary DNAs (cDNAs) were synthesized with 4 μg of total RNA, 0.4 μl random primer, 0.5 μl dNTPs, and 200 U MMLV reverse transcriptase, 5× RT buffer (4 μl) at 37 °C for 1 h, followed by 95 °C for 3 min, using a TaqMan Reverse-Transcription kit from Promega (Promega, Madison, WI). An aliquot of the resulting single-stranded cDNA was used in the PCR experiments. Based on the sequences reported in the GenBank database (Table 1), primers were designed for *Fbln1* and glyceraldehyde-3-phosphate dehydrogenase (*GAPDH*) with Primer Express Software (Applied Biosystems, Foster City, CA). Quantitative real-time PCR was performed with an ABI7500 Fast Real-Time PCR System (Applied Biosystems , Foster City, CA). A typical reaction was performed in 50 μl, consisting of 5 μl of cDNA and 10 μl of 5× SYBR Green I PCR buffer, containing the specific primer pairs (final 10 pmol each). The PCR temperature cycle was 93 °C for 3 min, followed by 40 cycles at 93 °C for 30 s, 55 °C for 45 s, and 72 °C for 45 s, with SYBR Green fluorescence recorded at the end of each elongation segment. The PCR reaction was followed by a melting curve analysis: 93 °C for 10 s, 55 °C for 10 s, linear increase to 93 °C at 0.1 °C/s, with continuous SYBR Green fluorescence recording. The primer pairs used in this study for *Fbln1* and *GAPDH* were validated as follows: they gave a single PCR product, as verified by melting curve analysis, agarose gel electrophoresis, and DNA sequencing; and the distribution of the PCR sigmoids was linear (r was 0.99 to 1) over 5 log units of template concentration with an efficiency of 1.85–1.98. The critical cycle of each sigmoid PCR curve was calculated by the ABI 7500 Fast Real-Time PCR System as the PCR cycle corresponding to the maximum of the second derivative. Total cDNA copy number from each cell culture sample was analyzed by the 7500 Fast Real-Time PCR Systems for *Fbln1* and *GAPDH*. All copy numbers from each sample were compared to the *GAPDH* cDNA copy number from corresponding samples.

**Table 1:** Primers used in real-time PCR reactions.

**Gene**	**GenBank accession**	**Primer sequence**	**Size (bp)**
H-*FBLN1*	NM_001996	Forward 5′-CATCAGCAGGATGTGTGTCGAT-3′ Reverse 5′-AGCGTGTTCTCGCACTTGTG-3′	81
H-*GAPDH*	NM_002046	Forward 5′-CCTGCACCACCAACTGCTTAG-3′ Reverse 5′-CAGTCTTCTGGGTGGCAGTGA-3′	111

### Western blot analysis

Western blot analysis was used to show the expression of fibulin-1 at the protein level. Briefly, cells were washed with PBS and lysed in ice-cold lysis buffer (Shanghai Xinghan Sci & Tech Co. Ltd, Shanghai, China). After cell debris was removed by centrifugation at 15,000g at 4 °C for 30 min, the protein concentration was detected by BAC kits (Shenneng Bocai Biotechnology Co. Ltd, Shanghai, China). Protein (40 μg) was loaded in each lane of 10% sodium dodecyl sulfate-polyacrylamide gels, transferred onto polyvinylidene difluoride membranes for electrophoresis, and blocked in Tris Buffered Saline with Tween (TBST) (5% fat-free dry milk, 0.1% Tween-20, 150 mM NaCl, and 50 mM Tris at pH 7.5) for 1 h. The membranes were exposed to 1:500 anti-fibulin-1 antibodies and incubated overnight at 4 °C. The same blots were then stripped and reanalyzed using anti-GAPDH antibodies (Beijing Biosynthesis Biotechnology Co. Ltd., Beijing, China) as an internal control. The membranes were then incubated with a secondary horseradish peroxidase (HRP)-labeled antibody (Beijing Biosynthesis Biotechnology Co. Ltd.) for 1 h. Protein bands were visualized with the use of a chemiluminescence Phototope (R)-HRP Western Blot detection system (Cell Signaling Technology, Inc., Danvers, MA) and exposed to a negative film, developed, and fixed. The film was scanned and analyzed with Quantity One Analyzer Software (Bio-Rad Laboratories, Santa Cruz, CA). The relative level of protein expression was expressed as the density ratio of the protein compared to GAPDH in the same sample. Independent experiments were performed and repeated three times.

### Statistical analysis

Data were expressed as the mean±standard deviation (SD) of at least three separate repeated experiments. Statistical analysis (SPSS version 12.0, SPSS, Chicago, IL, and SigmaPlot version 11, Chicago, IL) used one-way analysis of variance (ANOVA) or Kruskal–Wallis one-way analysis of variance on ranks as appropriate, with Tukey post hoc tests for all pair-wise comparisons where appropriate.

## Results

### Effects of gavage with retinoic acid on ocular size and refraction

Ocular size was increased by treatment with RA with a difference in axial length (axial distance from the cornea to the retina) of 97.5 µm (RA, 7.743±0.005 mm; PO, 7.628±0.009 mm, p<0.01), with most of the elongation due to an increase in the vitreous chamber depth (difference of 101.8 µm). For animals of this age, a 50-µm increase in ocular length is equal to approximately a −2D refractive shift [15]. We found here that RA treatment caused a myopic shift in refractive error of −4.8D (RA, 1.73±0.8D; PO, +6.5±1.6D, p<0.01).

### Expression of fibulin-1 and aggrecan in guinea pig sclera

**Figure 1:**
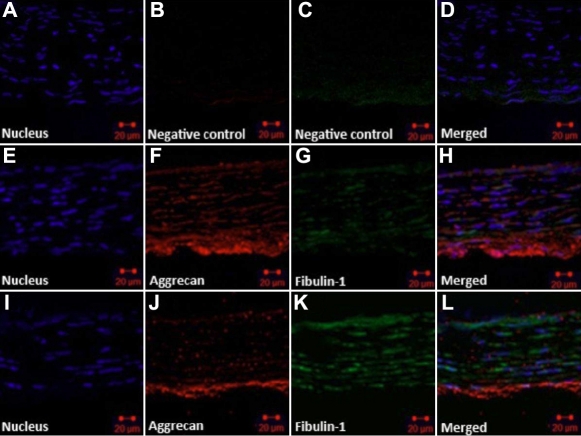
Expression and localization of fibulin-1 and aggrecan in guinea pig sclera. Pigmented guinea pigs were fed all-trans-retinoic acid (RA) or peanut oil (PO) for 5 days, then scleral sections were double labeled with antibodies for fibulin-1 (green) and aggrecan (red). Hoechst 33342 dyed the nucleus (blue: **A**, **E**, **J**). **A**–**D**: PBS was used instead of primary antibody as a negative control. Fibulin-1 was localized in scleral extracellular matrices in parallel with the distribution of aggrecan. **E**–**H**: Scleral tissue was taken from control animals fed vehicle (PO). Aggrecan was arranged between collagen fibrils and collagenous lamellae and dispersed in the outer layer of sclera. Fibulin-1 staining was weaker and surrounded several scleral fibroblasts. **I**–**L**: Scleral tissue was taken from animals fed RA. Aggrecan staining became weak, especially in the middle lamella, while fibulin-1 staining became substantially stronger. The original magnification was 400× and the scale bar=20 μm.

Fibulin-1 and aggrecan immunohistochemical staining were observed in scleras from RA- or PO-treated guinea pigs. In all eyes, fibulin-1 was localized in scleral extracellular matrices in parallel with the distribution of aggrecan. For the PO-fed guinea pigs, aggrecan was most strongly labeled in posterior sclera and was irregularly arranged between collagen fibrils and collagenous lamellae, with scleral fibroblasts located between them. In PO eyes, fibulin-1 immunoreactivity was low and was detectable in and around fibroblasts (Figure 1E–H). In RA-fed guinea pigs, the expression of fibulin-1 in the sclera was substantially increased, whereas the expression of aggrecan was depressed (Figure 1I–L).

### Effect of retinoic acid on the numbers of human scleral fibroblasts

**Figure 2:**
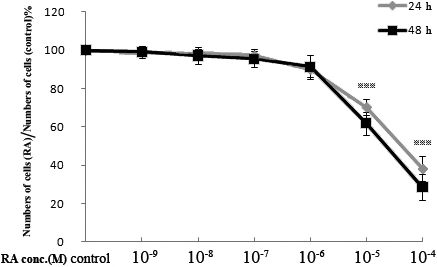
Effect of all-trans-retinoic acid (RA) on the numbers of cultured human scleral fibroblasts at 24 h or 48 h. Numbers of live cells were determined using the cell counting Kit-8. The results are shown as the numbers of live cells in the cultures with RA, as a percentage of the numbers in control cultures (mean±SD). Asterisks indicate significant differences when compared with the control without RA (p<0.001).

RA concentrations of less than or equal to 10^−7^ M did not change the number of live HSFs after 24 h or 48 h of co-culture (Figure 2). At higher concentrations of RA (greater or equal to 10^−5^ M), there were significant reductions in the number of live cells in comparison to control cultures. A small reduction in cell numbers also occurred with a concentration of RA of 10^−6^ M (10.1% at 24 h and 8.7% at 48 h), but these changes were not statistically significant. The half-maximal inhibitory concentration (IC_50_) of HSFs for RA was approximately 5×10^−5^ M.

### Changes in *Fbln1* mRNA levels in human scleral fibroblast cell cultures treated with retinoic acid

**Figure 3:**
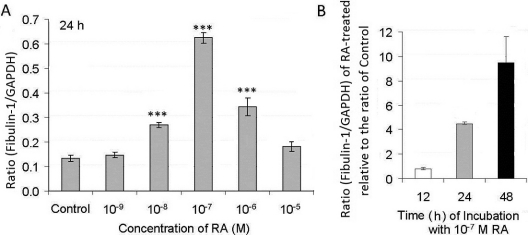
Effect of all-trans-retinoic acid (RA) on *Fbln1* mRNA levels in human scleral fibroblasts (HSFs). *Fbln1* mRNA levels were measured with real-time PCR analysis after cells were treated with various doses of RA. *Fbln1* mRNA abundance is expressed as cDNA copy numbers relative to copies of *GAPDH*. **A**: The ratio represents the ratio of *Fbln1* to *GAPDH* in control (0.1% DMSO) and treated HSFs after 24 h of incubation time with various doses of RA. Asterisks show significant differences relative to the appropriate control ratio (p<0.001). **B**: The ratio represents the ratio of *Fbln1* to *GAPDH* in HSFs with 10^−7^ M RA treatment compared to control after different incubation time (12, 24, and 48 h).The ratio of the RA is divided by the ratio of the respective control. Data are the mean±SD. Measures were repeated three times.

The effects of RA on *Fbln1* mRNA expression in HSFs were dose dependent (Figure 3). The *Fbln1* mRNA level in HSFs increased after treatment with RA for 24 h, with an RA concentration of 10^−7^ M giving the maximum increase (Figure 3A). Concentrations of RA that reduced cell numbers were less effective in the upregulation of *Fbln1* mRNA. To find the effective time that RA took to upregulate *Fbln1* mRNA expression in HSFs, total RNA prepared from cells treated with 10^−7^ M RA for different times (12 h, 24 h, 48 h) was analyzed and compared with total RNA from control cultures (Figure 3B). RA at 10^−7^ M induced the most marked expression of *Fbln1* mRNA in HSFs, and the effect was time dependent. There were no significant changes in *Fbln1* mRNA in HSFs after incubation with RA for 12 h, but *Fbln1* mRNA levels were significantly increased after treatment of HSFs with 10^−7^ M RA for 24 h and 48 h (p<0.001 in both cases), with the latter showing a dramatic increase of 9.4 times the control (Figure 3B).

### Retinoic acid-induced changes in fibulin-1 protein levels in cultured human scleral fibroblasts

**Figure 4:**
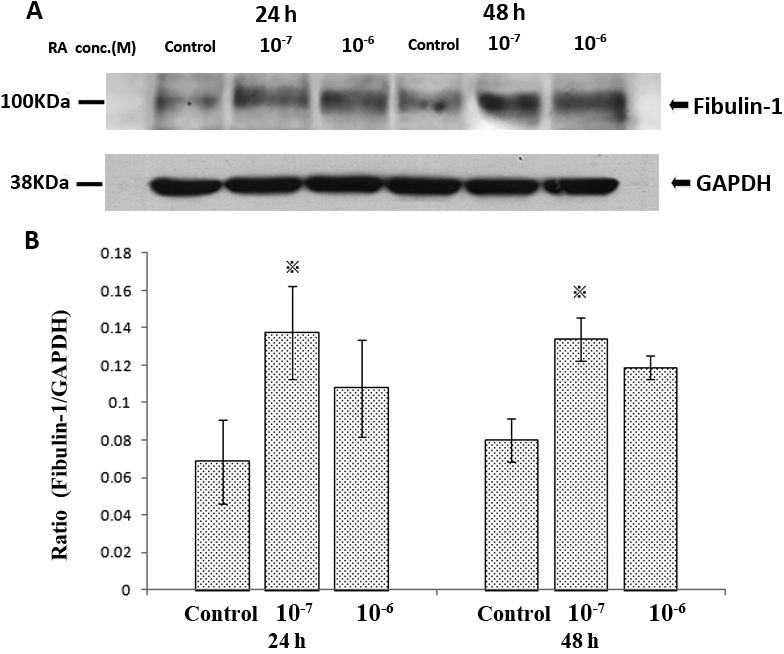
Effect of all-trans-retinoic acid (RA) for both 24h and 48h on the expression of fibulin-1 protein levels in human scleral fibroblasts (HSFs) using western blots. **A**: shows a western blot for fibulin-1 and GAPDH. No other bands were detected in the blot. Fibulin-1 was weakly expressed in control HSFs and significantly enhanced when co-incubated with 10^−7^ M of RA. **B**: Bar graphs show changes in protein expression (mean±SD, n=3) where density values were compared to GAPDH density. The asterisk indicates a significant difference relative to the control (p<0.05).

Protein prepared from the cells treated with RA concentrations of 10^−7^ or 10^−6^ M for either 24 h or 48 h were analyzed and compared with controls (medium with 0.1% DMSO). The relative protein levels of fibulin-1 in HSFs incubated with RA are represented in Figure 4. RA upregulated the fibulin-1 protein level in a time-dependent manner. The fibulin-1 protein expression was significantly increased after the cells were treated with RA at 10^−7^ M at both 24 h and 48 h (p<0.05).

### Morphological changes in human scleral fibroblasts and fibulin-1 immunohistochemical staining after retinoic acid treatment

**Figure 5:**
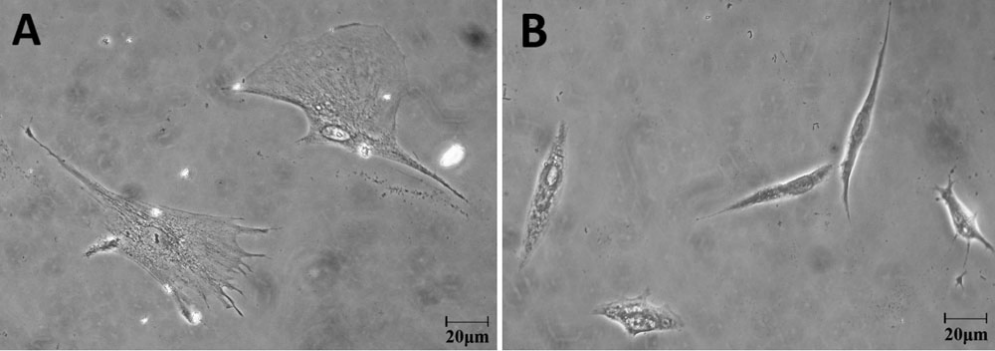
The effect of all-trans-retinoic acid (RA) on the morphology of cultured human scleral fibroblasts (HSFs) visualized with an inverted phase contrast microscope. **A**: In untreated control HSFs, cells spread on collagen I-coated standard plates and displayed many protrusions. **B**: After incubation with RA (10^−7^ M) for 48 h, the shape of the cells changed. The original magnification was 400× and the scale bar=20 μm.

**Figure 6:**
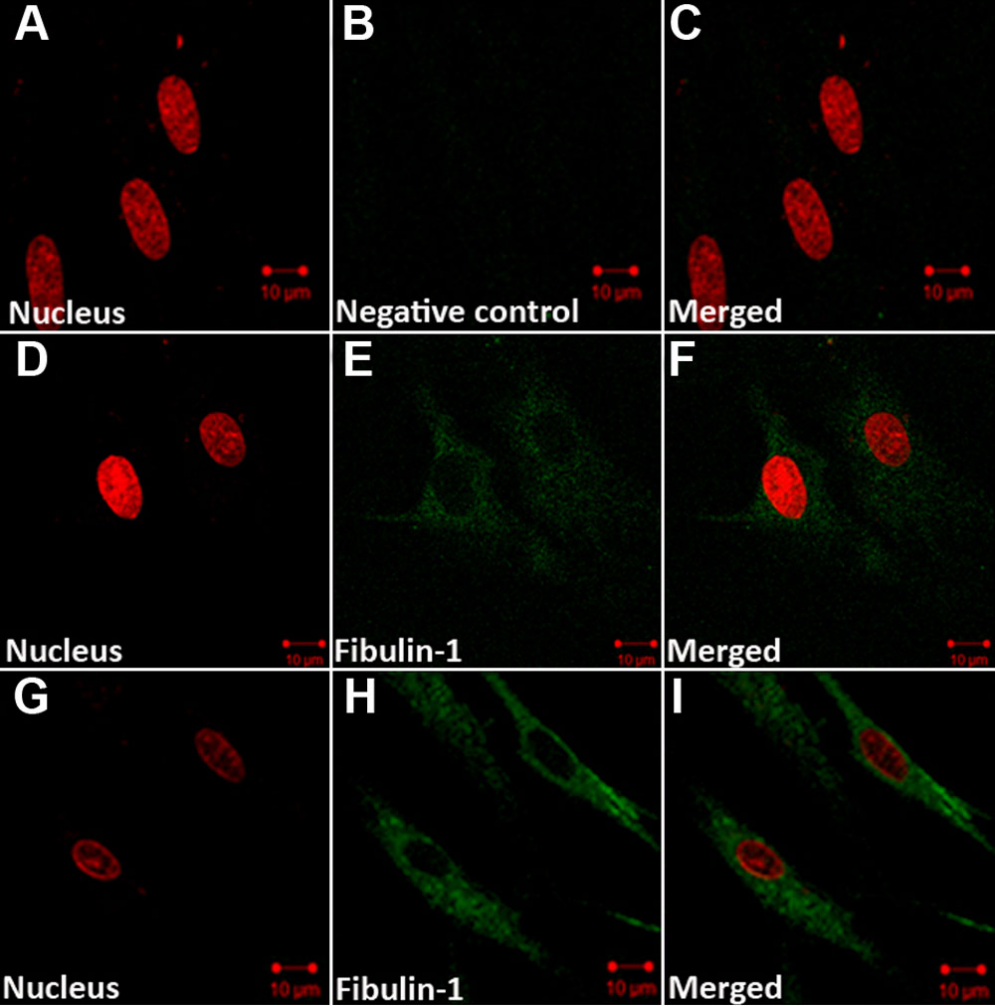
Expression of fibulin-1 in human scleral fibroblasts (HSFs) visualized by indirect immunofluorescence. The nuclei were stained with Propidium iodide dye (red: **A**, **D**, **G**), Dylight 488-conjugated secondary antibody was used for labeling with primary antibody (green: **B**, **E**, **H**). **A**–**C**: HSF cells were incubated in PBS without primary antibody as a negative control. **D**–**F**: HSFs were incubated in control medium. Fibulin-1 is weakly expressed in the cytoplasm. **G**–**I**: HSFs were incubated with retinoic acid (10^−7^ M) for 48 h. The expression of fibulin-1 in the cytoplasm of HSFs increased, and the morphology of HSFs changed. The original magnification was 1,000× and the scale bar=10 μm.

HSFs seeded onto collagen I and cultured in 10^−7^ M RA or medium with 0.1% DMSO (control) for 48 h showed morphological changes. Under the inverted phase contrast microscope, control HSFs displayed many spreading protrusions on collagen-coated standard plates (Figure 5A). After RA treatment HSFs became smaller with little evidence of any protrusions (Figure 5B). With fibulin-1 immunohistchemistry, fibulin-1 was found to be weakly expressed in the cytoplasm of control cells with a uniform distribution (Figure 6D–F). In cells cultured in a medium containing 10^−7 ^M RA for 48 h, fibulin-1 expression was significantly increased (Figure 6G–I).

## Discussion

Fibulin-1, which is an important ECM molecule, is associated with tissue remodeling in many systems [1,2]. Recent work has shown that fibulin-1 is expressed in human sclera [7]. Here, we provide evidence that fibulin-1 protein is expressed in vivo in guinea pig sclera and that gavage with RA leads to increased expression of fibulin-1 and decreased expression of aggrecan. We further show that fibulin-1 is expressed in HSFs grown in culture, where expression is also regulated by RA. These results suggest that RA regulates the expression of fibulin-1 similarly in vivo and in vitro and that the molecular pathways involved may therefore be further studied using cultured fibroblasts.

RA can affect scleral ECM during scleral remodeling [10]. RA appears to regulate the expression of aggrecan in skeletal systems and affect cartilage matrix homeostasis [11]. However, the effect of RA on aggrecan and fibulin-1 in the sclera has not been previously reported. In this study we fed RA to normal guinea pigs, and the eyes rapidly elongated and became myopic. Using immunohistochemical staining, we found that fibulin-1 and aggrecan were distributed in a similar fashion in normal guinea pig sclera, but fibulin-1 staining was weak, whereas aggrecan staining was stronger. In the sclera from RA-fed animals, the expression of fibulin-1 was increased, while there was a loss of scleral aggrecan staining. Similar results have been reported on cultured cartilage tissue where exposure to RA led to the extensive loss of aggrecan from the tissue [16]. The reciprocal relationship between fibulin and aggrecan observed in our study may be due to the ability of fibulin-1 to enhance the cleavage of aggrecan by a disintegrin-like and metalloprotease with thromobospondin type-1 [6,17]. This suggests that the pathway leading to increased eye growth may involve upregulation of fibulin-1 by RA and degradation of aggrecan.

The expression of fibulin-1 was induced by RA in a dose-dependent manner in cultured HSFs, as shown by both real-time PCR and western blot analysis. A 10^−7^ M concentration of RA maximally increased the expression of fibulin-1 protein in HSFs. This concentration is similar to the concentration of RA shown to inhibit proteoglycan synthesis in cultured chick sclera [10], and we have shown here that this concentration does not reduce the number of cells that grow under cultured conditions. Since RA appears to be an important molecular signal in the control of eye size [9,10,18-20], it is tempting to relate the capacity of RA to inhibit aggrecan synthesis in cultured sclera to its effect on eye growth.

Although the functions of fibulin-1 in the sclera remain unclear, our finding of fibulin-1 expression induced by RA, which is a molecule known to be involved in the regulation of eye growth, suggests that it is worth looking at the role of fibulin-1 in scleral remodeling. Interactions between fibulin-1 and aggrecan may be important, and the modulation of aggrecan levels and distribution could play a key role in changing the scleral creep rate [5], which is associated with axial elongation of the eye and the development of myopia [21]. Further research on the function of fibulin-1 and its interaction with aggrecan in sclera during the development of myopia is warranted.
